# Coma

**Published:** 1871-10

**Authors:** Robert Battey

**Affiliations:** Rome, Ga.


### COMA.

By Robert Battey, M. D., Rome, Ga.

Case I.—Mr. M——, 60 years of age, Rome, Ga., long dyspeptic, in his usual rather feeble health, ate at dinner on the 6th of August, 1865, rather mature boiled corn cut from the cob with a knife, and improperly masticated because of deficient teeth. On the 7th he felicitated himself upon his exemption from the troublesome symptoms of indigestion, which he had anticipated, as the penalty of his indiscretion. At night he retired early, not feeling very well, but making no special complaint; slept well and soundly, so much so, indeed, that he did not wake at his usual hour in the morning. His wife observing this endeavored to arouse him, but unsuccessfully, and called in aid. He was seen about sunrise.

There were no special symtoms, except the Coma which was deep and lethargic. He was profoundly insensible to every external impression, whether of sound, of light, or of the infliction of pain; all deglutition was impossible, a large supply of strong red pepper tea was ordered at once; a portion of it to be thickened with corn meal for poultices. The feet and legs were promptly immersed in a bath of *hot* water, at a temperature considerably more elevated than usual for baths; two pints of pepper tea, as hot as the bath, was thrown into the bowels with a syringe; and a large pepper-poultice, of like temperature, covered the abdomen. With this combination of bath, enema, and poultice, highly stimulating, both by irritation and temperature, there was no sign of returning conciousness, no evidence of sensibility, no movement of muscle.

The treatment was steadily continued; hour after hour the poultices were renewed, as temperature subsided, until the hands of the attendant were as red as lobster claws; and yet there was not even a blush upon the abdomen of the patient. The pepper enema was retained; twice repeated, and still retained; the strength of the tea being each time increased. The feet scalded in the hot baths like blocks of wood, and seemed as little affected.

About 4 p. m., dim signs of sensibility were noted; soon the patient slided towards the edge of the bed and raised himself up. Assisted by nurses, he stood upon the floor, as rigid as a post, and equally unconcious of all that was passing around him. In this position the bowels suddenly expelled upon the floor, the copious enemata, together with a pint or more of the grains of corn, unaltered, as when eaten fifty-two hours previously.

Conciousness now rapidly returned; by 9 p. m., he was entirely rational, expressed himself as quite comfortable, drank a little tea, took a purgative, and rested well the night. Wednesday the 9th, he was bright; sat up in bed; ate a light breakfast; thought he could put on his clothes; doubted whether he would need any further medical attention. The purgative had acted well; nothing more was given.

Thursday, 10th, complained of headache; pulse a little accelerated; slight fever. On the 11th, there was more fever, and constant delirium. On the 12th, Coma returned; collapse, then death. There was no post-mortem.

CASE 2.—Mr. G., age 30 years, married, dyspeptic, in usual health, ate hard boiled eggs for dinner, on the first November, 1866; a diet of which he was very fond, but which had uniformly given him attacks of indigestion. At 10 p. m., he returned, feeling entirely well, and boasted of his immunity from anticipated suffering. An hour later, against the remonstrances of his wife, he rose and ate two more of the eggs, left from dinner. In the morning, he could not be awakened, but had no other alarming symptoms. The feet and legs were put into scalding water, which produced some muscular movements, evincing slight sensibility to pain, but nothing more. Repeated hot enemata of pepper tea, with hot fomentations of pepper to the abdomen, moved the bowels and restored conciousness in about three hours; when a dose of cathartic pills emptied the bowels completely, and convalescence was promptly established.

CASE 3.—Mrs. R., 32 years old, married, mother of several children, suffered for some years with chronic endometritis; rather hysterical. Living some distance in the country, she was attacked by chill, with hysterical paroxysms, on the 9th of May, 1868. She became greatly alarmed for her safety; fancied she was dieing; took, at intervals, a pint or more of strong brandy, and sank into a state of most profound Coma.

When seen, a little after dark, the insensibility was deep and universal; pulse small and feeble; breathing stertorous. The feet and legs were dropped into *scalding* water, in which the hand could not be comfortably borne for a moment; there was no sign of sensibility. An enema of very hot tea, was thrown into the rectum, through a sphincter so perfectly relaxed and open, the fluid trickled back upon the bed, like water from a capsized bottle. Hot irritating fomentations were plied upon the abdomen assiduously, and the bath renewed, from time to time, until muscular movements were evident in the legs. The patient drew her feet slowly out of the tub, but they were at once replaced, and kept in the scalding water, until returning conciousness enabled her to call out boisterously for relief from torture. An emetic was now given, and portions of the brandy vomitted, when she sank into a quiet natural sleep.

In the morning she was every way comfortable, excepting that the limbs were badly bruised, where she had been slapped and beaten by the friends, in their vain efforts to arouse her. It was remarkable that the scalding water left no signs of redness or irritation upon the feet and legs. There was no exfoliation; not even any discomfort complained of from this cause. Neither in this nor the preceeding case was there any irritation, sense of heat, or inconvenience in any way, produced by the strong pepper injections.

Case 4.—Mrs. G., 46 years of age, vesico vaginal fistula, of twenty-two years standing; twice unsuccessfully operated upon by Drs. Francis and Stevens, of New York. Operated in the fall of 1863, assisted by Surgeon L T. Pim, P. A. C. S. Patient stated, when put to bed, that she could not take morphine or other opiate, but it was deemed so important that her nervous system should be thoroughly quieted, that a half grain of the sulphate morphine was dissolved in an ounce of water, and thrown into the bowels, without her knowledge.

Summoned in the greatest haste, an hour and a-half afterwards, the symptoms seemed most alarming. The face was livid; "blue as an indigo bag;" respiration very slow and stertorous, puffing from the lips like a pipe smoker; pulse thirty-eight, and feeble; Coma amounting to the deepest lethargy.

It was at once apparent that no time was to be lost. From a physician's office across the street, was quickly obtained a large syringe and a pound bottle of tincture of capsicum. The syringe was filled, and the bowels injected with the undiluted tincture; which remained in the canal for more than an hour; when it was withdrawn, as well as could be, through a large gum catheter, and a quart of strong infusion of coffee thrown in and retained. In the mean time, mustard frictions to the extremities, and hot pepper fomentations to the abdomen, were dilligently used.

In four hours' time, sensation and conciousness gradually returned. She passed a good night; was very comfortable in the morning, and went on perfectly well to a happy result of the surgical operation. She was watched for days, for signs of rectal inflammation, which were confidently looked for; but so far from this, the bowels remained unmoved the full ten days, and were not complained of in the slightest degree. She was not even aware that the bowels had been interfered with in any way.

*Remarks.*—A point worthy of note, in the two first cases, is that the gastric irritation produced in dyspeptic stomachs by indiscretion in diet, failing to manifest itself in an ordinary way, by the usual gastric symptoms, explodes itself, so to speak, upon the brain, and profound Coma is the result. It is probable that had either of these patients suffered the sour stomach, gastrodynia, etc., to which they were both accustomed, on such occasions, the brain would have escaped unharmed.

A second point of interest is the fact that, in this state of profound Coma, irritants of an otherwise intolerable power, are borne with impunity. Scalding water does not scald; cayenne pepper does not burn. This is not alone relatively true, as regards the sensations of the patient, which are for the time in abeyance, but it is absolutely true, that the hands of the attendant are reddened and irritated most painfully, while the more tender surfaces of the patient are not in the least affected. Even a delicate mucous membrane bears the contact of strong tincture capsicum for an hour, without inflammation, irritation, pain, or the least discomfort.

The third and last point. There is a power in the *bold use* of these very simple and ever-ready means, to arouse the powers of life in extreme cases, which ought to be duly pondered in every nook and corner of our wide-spread land.

